# 

**Published:** 1903-06

**Authors:** 


SUBSCRIPTION $1.00.

PUBLISHED MONTHLY

### ATLANTA JOURNAL-RECORD OF MEDICINE

Successor to Atlanta Medical and Surgical Journal, Established 18[illegible], and Southern Medical Record, Established 1870.

VOL. V. ATLANTA, GA., JUNE, 1903. NO. 3.

